# Impact of Subarachnoid Hemorrhage on the Cardiac Autonomic Function During Rehabilitation in Children After Severe Traumatic Brain Injury

**DOI:** 10.1089/neur.2023.0032

**Published:** 2023-07-14

**Authors:** Gilad Sorek, Sharon Shaklai, Isabelle Gagnon, Kathryn Schneider, Mathilde Chevignard, Nurit Stern, Yahaloma Fadida, Liran Kalderon, Michal Katz-Leurer

**Affiliations:** ^1^ Department of Physical Therapy, Faculty of Medicine, Tel Aviv University, Tel Aviv, Israel.; ^2^ Children Rehabilitation Department, Lowenstein Rehabilitation Center, Ra'anana, Israel.; ^3^ Montreal Children's Hospital, McGill University Health Center, Faculty of Medicine and Health Science, McGill University, Montreal, Quebec, Canada.; ^4^ School of Physical and Occupational Therapy, Faculty of Medicine and Health Science, McGill University, Montreal, Quebec, Canada.; ^5^ Sport Injury Research Prevention Centre, Faculty of Kinesiology, University of Calgary, Calgary, Alberta, Canada.; ^6^ Alberta Children's Hospital Research Institute, University of Calgary, Calgary, Alberta, Canada.; ^7^ Hotchkiss Brain Institute, University of Calgary, Calgary, Alberta, Canada.; ^8^ Sorbonne Université, Laboratoire d'Imagerie Biomédicale, LIB, Inserm, CNRS, Paris, France.; ^9^ Sorbonne Université, GRC 24 HaMCRe, Paris, France.; ^10^ Rehabilitation Department for Children with Acquired Neurological Injury, Hôpitaux de Saint Maurice, Saint Maurice, France.; ^11^ Alyn Children's Hospital and Rehabilitation Center, Jerusalem, Israel.

**Keywords:** cardiac autonomic control system, children and adolescents, rehabilitation, subarachnoid hemorrhage, traumatic brain injury

## Abstract

This study aimed to investigate the impact of traumatic subarachnoid hemorrhage (tSAH) on cardiac autonomic control system (CACS) function in children after severe traumatic brain injury (TBI) during the subacute rehabilitation period. Thirty-three participants, 8–18 years of age, 42 (14–149) days after severe TBI at the beginning of the subacute rehabilitation, were included in the study. Six participants were diagnosed with tSAH during acute medical care (tSAH group). Heart rate variability (HRV) was assessed by the standard deviation of the N-N interval (SDNN) and the square root of the mean square differences of successive R-R interval (RMSSD) using a Polar RS800CX device while sitting at rest for 5 min. A second assessment was performed 8 weeks later. No significant difference between the tSAH and non-tSAH groups were found in the demographic and functional characteristics or injury severity. However, in comparison to the non-tSAH group, the tSAH group had lower SDNN (23.9 [10.5–47.3] vs. 43.9 [21.8–118.8], respectively; *p* = 0.005) and RMSSD values (11.8 [5.9–24.5] vs. 29.6 [8.9–71.7], respectively; *p* = 0.004). Neither group demonstrated changes in HRV values at rest in the second assessment, whereas the significant difference in SDNN (*p* = 0.035) and RMSSD (*p* = 0.008) remained. Children diagnosed with SAH after severe TBI presented poorer CACS function during the subacute rehabilitation. Given that reduced HRV values may be a marker for potential heart disease, the medical team should be aware of the influence of existing tSAH. Future studies with larger sample sizes and longer follow-up periods are warranted to further investigate this topic.

ClinicalTrials.gov number: NCT03215082

## Introduction

Traumatic brain injury (TBI) is the most common cause of acquired disability in children and young adults.^1–3^ Pediatric TBI incidence rates vary widely by country, ranging from 47 to 280 per 100,000 person-years, with moderate-severe TBI accounting for ∼20% of TBIs.^3^ An impaired cardiac autonomic control system (CACS) is frequently observed post-TBI.^1,4^ CACS function may be assessed non-invasively using heart rate variability (HRV) parameters. HRV reflects beat-to-beat changes in RR intervals and represents the interaction between sympathetic and parasympathetic systems on the heart.^5^ Reduced HRV values are associated with increased mortality risk and may be a marker for heart disease.^6,7^ During subacute rehabilitation, children after severe TBI exhibit impaired CACS function and reduced HRV parameters.^8^

Subarachnoid hemorrhage (SAH) injury may also produce autonomic dysfunction, and pathophysiological overlap exists between central hypersympathetic dysfunction and the major SAH complications.^9^ Patients after aneurysmal SAH exhibit an acute hypersympathetic state,^10^ and ∼20% of patients experience cardiac dysfunction.^10^

The possible impact of traumatic SAH (tSAH) on CACS function is not well understood. This report aims to present the impact of SAH on CACS function in children after severe TBI during the subacute rehabilitation period.

## Methods

This study is a secondary analysis of a planned substudy of the SiMPLy-Rehab study. Participants were enrolled between August 2018 and December 2021 at the Alyn Children and Adolescent Rehabilitation Hospital and Loewenstein Rehabilitation Center, Israeli sites of the study. The study was registered with the Clinical Trials Registry (#NCT03215082) and was approved by the Helsinki ethics committees of both hospitals and the ethics committee of Tel Aviv University. Verbal informed consent was obtained from each child and their parents, who also gave written consent before their child participated.

The study included 33 children, 8–18 years of age, after severe TBI (Glasgow Coma Scale [GCS] at the time of injury <9 and/or loss of consciousness [LOC] >24 h^11^). Children entered the study when their medical condition stabilized and their physician cleared them to initiate rehabilitation. Exclusion criteria were previous brain injury, known heart disease, medications that affect cardiac autonomic function (e.g., beta-blockers, alpha agonists, and methylphenidate), previous orthopedic, psychiatric, or neurological problems, blindness, deafness, or spinal cord injury.

Diagnosis of SAH was based on computerized tomography or magnetic resonance imaging, performed during the acute hospitalization after injury and collected from each participant's medical file.

Heart rate and HRV values were recorded using a Polar RS800CX watch and chest strap, with a sample rate of 1000 Hz (Polar Electro OY, Kempele, Finland). HRV time measures included the SDNN (standard deviation of the N-N interval) and the RMSSD (the square root of the mean square differences of successive R-R interval).^5,12^ Frequency measurements of HRV include the band in the range between 0.04 and 0.15 Hz (low frequency; LF), the band in the range between 0.15 and 0.40 Hz (high frequency; HF), and the relationship between LF and HF (LF/HF).^5,12^ After the recording, the data were transferred to a computer, visually reviewed, and filtered. HRV measures were assessed during rest in supported sitting for 5 min.

Assessment of motor function was completed using the motor section of the Functional Independence Measure (FIM).^13^

Participants were asked to avoid eating an hour before the assessment and to go to the bathroom before the assessment began. The evaluation was performed between 12:00 pm and 5:00 pm in a quiet treatment room maintained at a temperature of 20°C–24°C. A trained physiotherapist performed all assessments. Background variables (demographic, anthropometric, medical, and functional characteristics) were collected from the medical records before the evaluation. During the assessment, participants were asked to rest in a supported sitting position for 5 min while wearing a chest strap that collected data. The second assessment was performed after 8 weeks of standard rehabilitative treatment for children after severe TBI.^1^

Because of the small number of participants with SAH, non-parametric tests were used. Differences between groups were tested using Fisher's exact test or Mann-Whitney's U test. Differences within groups were tested using the Wilcoxon test. A rigorous approach was taken for the follow-up analysis, and the follow-up time point analysis included all participants after TBI, including the children who dropped out (*n* = 4); the values of the first assessment were carried forward to the second assessment. Spearman's correlation coefficients were used to evaluate associations between background variables and HRV measures. Alpha level was set *a priori* at *p* < 0.05. Statistical analyses were done using the SPSS software packages (version 24; SPSS, Inc., Chicago, IL).

## Results

Children were initially assessed between 2 weeks and 6 months after injury. Based on medical records, 6 of the 33 children who participated in the study were diagnosed with tSAH, whereas the remaining 27 children were included in the non-SAH group.

Demographic, anthropometric, and injury severity characteristics and functional abilities upon admission for both groups are presented in Table 1. No significant differences were found between the SAH and non-SAH groups for any of the parameters. GCS at time of injury was not presented because of missing values for most SAH-group participants (*n* = 5).

**Table 1.** Participant's Characteristics at the First Assessment for Patients With and Without Traumatic SAH

Characteristic	SAH group (*n* = 6)	Non-SAH group (*n* = 27)	*p *value
Age (years)	16.7 [9.9–17.8]	14.1 [8.0–18.4]	0.40
Sex (female/male)	0/6	3/24	1.00
Body mass index (kg/m^2^)	19.3 [13.2–32.4]	17.3 [13.8–25.7]	0.56
LOC (days)	8 [4.0–60.0]	6 [0.25–30.00]	0.40
Time post-injury (days)	34 [25–117]	43 [14–149]	0.80
FIM Transfers (/7)	5 [3–7]	5 [2–7]	0.66
FIM Locomotion (/7)	5 [1–6]	5 [2–6]	0.91
FIM Stairs (/7)	5 [1–6]	5 [1–6]	0.91

Values: numbers, median [minimum-maximum]; *p* value: Fisher's exact test/Mann-Whitney U test.

SAH, subarachnoid hemorrhage; LOC, loss of consciousness; FIM, Functional Independence Measure.

Two children dropped out from each group before the follow-up assessment. One chose not to continue the study, and 3 completed the rehabilitation before the second assessment and were discharged before the follow-up testing session.

CACS values at the first and second assessments are presented in Table 2. Results indicate that the SAH group exhibited markedly lower median HRV values than the non-SAH group at baseline and 2-month follow-up, except for the LF/HF value. For example, regarding time-domain HRV measures, the SAH group displayed a median RMSSD value of 11.8 ms (5.9–24.5) during the first assessment, which was significantly lower than the non-SAH group's 29.6 ms (8.9–71.7; *p* = 0.004). This difference persisted even after 8 weeks, with the SAH group recording 18.9 ms (13.0–22.4) and the non-SAH group 25.2 ms (5.5–91.2; *p* = 0.008). When examining the frequency-domain HRV measures, the same trend was observed with HF values. The median HF value for the SAH group was 53 ms^2^ (7–313), whereas for the non-SAH group it was 462 ms^2^ (19–1701; *p* = 0.011). During the second assessment, the SAH group presented a median HF value of 128 ms^2^ (74–232), whereas the non-SAH group presented a median HF value of 360 ms^2^ (19–2314; *p* = 0.007).

**Table 2.** Cardiac Autonomic Control System Function at Rest While Sitting in the Group With (*n* = 6) and Without SAH (*n* = 27)

	First assessment			Second assessment			Change over time* p *value		
	SAH	Non-SAH	*p *value	SAH	Non-SAH	*p *value	SAH	Non-SAH	Between groups
HR (BPM)	105 [90–116]	88 [68–112]	**0.040**	96 [89–116]	91 [68–112]	0.205	0.273	0.435	0.260
SDNN (ms)	23.9 [10.5–47.3]	43.9 [21.8–118.8]	**0.005**	34.6 [23.4–40.3]	43.6 [14.0–83.4]	**0.035**	0.138	0.407	0.132
RMSSD (ms)	11.8 [5.9–24.5]	29.6 [8.9–71.7]	**0.004**	18.9 [13.0–22.4]	25.2 [5.5–91.2]	**0.008**	0.345	0.123	0.145
LF (ms^2^)	212 [59–622]	642 [141–3881]	**0.004**	336 [223–524]	727 [66–2475]	**0.013**	0.345	0.607	0.665
HF (ms^2^)	53 [7–313]	462 [19–1701]	**0.011**	128 [74–232]	360 [19–2314]	**0.007**	0.893	0.331	0.538
LF/ HF	3.5 [1.4–7.7]	1.7 [0.5–7.3]	0.089	3.1 [1.8–3.5]	2.2 [0.7–6.7]	0.451	0.500	0.607	0.348

Bolded values: significant between groups difference, *p* value < 0.05.

Values: median [minimum-maximum]; *p* value: Mann Whitney U test/Wilcoxon test.

SAH, subarachnoid hemorrhage; HR, heart rate; BPM, beat per minute; SDNN, standard deviation of the N-N interval; RMSSD, root mean square of successive differences; LF, low frequency; HF, high frequency.

No significant change in HRV measures was noted over time in each group (Table 2).

No associations were found between HRV variables and LOC in days, time post-injury, age, body mass index, or functional level (according to the FIM).

No cardiac adverse event occurred throughout the study period for both groups.

## Discussion

Children diagnosed with SAH after severe TBI presented lower HRV values compared to those without SAH. These differences persisted after 8 weeks of rehabilitation. Further, the diagnosis of SAH was the only variable significantly associated with the HRV measures.

The impaired CACS function observed subsequent to severe TBI can be attributed to the extent and severity of brain injury. Neural CACS function is processed in several brain centers, including the medulla, hypothalamus, insular cortex, cingulate cortex, and pre-frontal cortex.^12,14^ Severe brain damage and impaired central information processing can affect the CACS's ability to respond and adapt over time.^8,15,16^ tSAH can exacerbate the hemodynamic-metabolic crisis within the brain, resulting in additional cardiac impairment. This can lead to even more profound impairment of CACS function during the subacute rehabilitation period.

This study has several limitations, with the most significant being the small sample size of the SAH group and the relatively short follow-up period. In addition, there were very few female participants in this study, none with SAH. Therefore, future studies with larger sample sizes and longer follow-up periods are needed to gain a better understanding of the presence and progression of CACS dysfunction in this population and better understand differences in response between males and females.

## Conclusion

These preliminary findings indicate that children diagnosed with SAH after severe TBI had lower HRV values than those without SAH. Given that reduced HRV values may be a marker for heart disease,^6,7^ the results of this study emphasize the need for CACS assessment in children after severe TBI. Moreover, medical professionals caring for persons with severe TBI should be aware of the potential impact of tSAH on CACS function and the need for appropriate monitoring and treatment.

## Abbreviations Used

CACS: cardiac autonomic control system
FIM: Functional Independence Measure
GCS: Glasgow Coma Scale
HF: high frequency
HRV: heart rate variability
LF: low frequency
LOC: loss of consciousness
RMSSD: root mean square of successive differences
SAH: subarachnoid hemorrhage
SDNN: standard deviation of the N-N interval
TBI: traumatic brain injury
tSAH: traumatic subarachnoid hemorrhage
